# Integrating modality-specific expectancies for the deployment of spatial attention

**DOI:** 10.1038/s41598-018-19593-7

**Published:** 2018-01-19

**Authors:** Paola Mengotti, Frank Boers, Pascasie L. Dombert, Gereon R. Fink, Simone Vossel

**Affiliations:** 1 Cognitive Neuroscience, Institute of Neuroscience & Medicine (INM-3), Research Centre Juelich, 52425 Juelich, Germany; 2 Institute of Neuroscience & Medicine (INM-4), Research Centre Juelich, 52425 Juelich, Germany; 3 Department of Neurology, University Hospital Cologne, 50937 Cologne, Germany; 4 Department of Psychology, University of Cologne, 50923 Cologne, Germany

## Abstract

The deployment of spatial attention is highly sensitive to stimulus predictability. Despite evidence for strong crossmodal links in spatial attentional systems, it remains to be elucidated how concurrent but divergent predictions for targets in different sensory modalities are integrated. In a series of behavioral studies, we investigated the processing of modality-specific expectancies using a multimodal cueing paradigm in which auditory cues predicted the location of visual or tactile targets with modality-specific cue predictability. The cue predictability for visual and tactile targets was manipulated independently. A Bayesian ideal observer model with a weighting factor was applied to trial-wise individual response speed to investigate how the two probabilistic contexts are integrated. Results showed that the degree of integration depended on the level of predictability and on the divergence of the modality-specific probabilistic contexts (Experiments 1–2). However, when the two probabilistic contexts were matched in their level of predictability and were highly divergent (Experiment 3), higher separate processing was favored, especially when visual targets were processed. These findings suggest that modality-specific predictions are flexibly integrated according to their reliability, supporting the hypothesis of separate modality-specific attentional systems that are however linked to guarantee an efficient deployment of spatial attention across the senses.

## Introduction

Deploying attention in space is a flexible process that enables us to react efficiently to environmental stimuli. In cueing paradigms, in which a cue predicts the location of a target with a specific probability, valid cues lead to faster detection of the target, whereas invalid cues cause slower response times^1^. Recent evidence suggests that the deployment of attention is modulated by hidden changes in cue predictability, i.e., by the probability that the cue validly predicts the target location^2–4^. Higher cue predictability resulted in higher response speed differences between validly and invalidly cued trials. By applying computational models to behavioral data, these studies have shown that subjects track trial-wise estimates of cue predictability and adapt their behavior accordingly. As for the neural correlates, the right temporo-parietal junction was consistently involved in processing cue predictability in spatial attention tasks^2–4^.

The majority of studies used cueing paradigms within one sensory modality only and the visual modality has been studied most thoroughly. However, some studies have investigated spatial attention with targets in different modalities^5–8^. In a series of behavioral studies, Spence *et al*.^8^ showed that expectations about the location of a target in one modality (either visual or tactile) also modulated expectations in the other modality, arguing for the existence of crossmodal links between vision and touch. Their data suggest that there is some degree of integration or merging of concurrent but divergent modality-specific cue predictability. At the same time, the findings were not compatible with an entirely supramodal account of spatial attention, since differences in cueing effects were still present for the two modalities. Nevertheless, imaging studies have provided evidence of potentially supramodal effects^5,6^. In a multisensory cueing paradigm performed during functional MRI^6^, regions of the ventral attentional system^9^, i.e., the temporo-parietal junction and the inferior frontal cortex, were activated during attentional reorienting, both for visual and tactile targets. Furthermore, anticipatory attentional shifts induced the same modulation of ERP components independently from target modality^5^ (i.e., vision or touch). In contrast, results of a transcranial magnetic stimulation (TMS) study^10^ challenged the idea of a supramodal organization of attention by showing that disruption of the activity of the right supramarginal gyrus impaired cueing effects for visual but not for tactile targets. Taken together, these findings show that strong crossmodal links exist in the deployment of spatial attention. At the same time, they argue against an unconditional supramodal nature of the effects, but it remains unclear which factors influence the degree of integration.

In this series of behavioral experiments, we used computational modelling of behavior to investigate if and under which circumstances crossmodal links during the deployment of spatial attention occur. We employed a multisensory version of a location-cueing paradigm, similar to the task employed by Macaluso *et al*.^6^, in which an auditory cue predicted the location of a visual or tactile target with modality-specific predictability, thereby creating two concurrent probabilistic contexts. For example, within an experimental block, the cue predictability could be high for visual targets, predicting their location correctly most of the time, but it could be low for tactile targets. We applied a Bayesian ideal observer model in each modality and investigated how the two different model-derived cue predictabilities were weighted in different experimental blocks. This weighting factor was estimated from observed response speed to disambiguate between three alternative hypotheses: if attentional systems are fully supramodal, participants should merge the two modality-specific cue probabilities into an average prediction by giving equal weight to both model-derived cue predictabilities. Conversely, if attention is deployed independently in different modalities, the behavior of the participants in the two modalities should be guided separately by the respective modality-specific cue predictability. Thirdly, participants may flexibly weigh the two concurrent cue predictabilities depending on their absolute level as well as on their divergence.

In Experiment 1, participants were not informed whether a visual or tactile target was going to appear, requiring them to simultaneously prepare for both types of targets. In Experiment 2, we aimed at eliminating this uncertainty by cueing the target modality with 100% predictability, before the appearance of the probabilistic spatial cue. Participants were hence always informed about the modality of the target and were required to prepare only for one target depending on the modality-specific probabilistic context. In Experiment 3, we made the two modalities equally predictive within each block, but in opposite directions to increase the divergence of the two cue predictabilities. For example, when the cue predictability was 90% for visual targets and 10% for tactile targets, the level of predictability of the cue was the same (when considering the distance from 50%), but with an inverted proportion of valid and invalid trials. We hypothesized that in this case participants would use a more separated processing of the two modality-specific probabilistic contexts.

## Methods

### Participants

Twenty-one healthy volunteers (18–32 years; 11 females) participated in Experiment 1. Twenty-one healthy new volunteers (19–39 years; 10 females) participated in Experiment 2. Twenty-one healthy volunteers (18–33 years; 12 females) participated in Experiment 3, of which five already participated in one of the two previous experiments. They were all German native speakers and had normal or corrected to normal vision, no past or present neurological or psychiatric condition, and they were naïve to the purpose of the experiment. All participants were right-handed, as assessed with the Edinburgh Handedness Inventory^11^. The study had been approved by the ethics committee of the German Psychological Society and was performed in accordance with the Code of Ethics of the World Medical Association (Declaration of Helsinki). Informed consent was obtained from all participants.

### Stimuli and experimental paradigm

**Figure 1:**
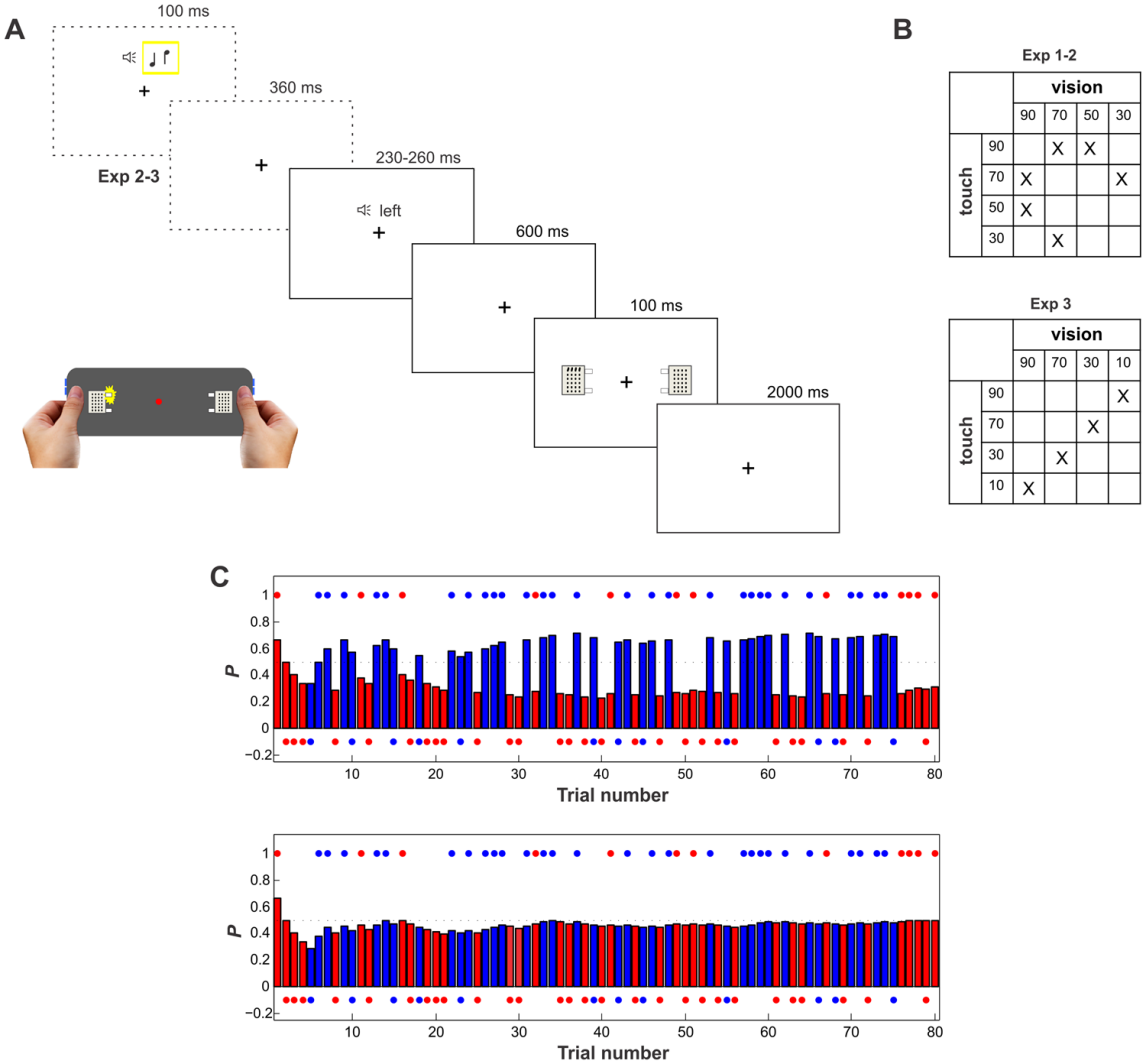
Experimental paradigm and illustration of the modeling approach. (**A**) Timeline of the experimental paradigm for a validly cued tactile trial. On each trial, participants indicated whether a distal or a proximal stimulus had been presented. Central fixation had to be maintained throughout the experiment. In Experiment 1 only the spatial cue was presented. In Experiment 2–3 an additional cue indicated the target modality (dashed frames). In the bottom part of the figure the multisensory device is represented, showing a visual stimulation. Participants kept their thumbs on the tactile stimulators and LEDs were placed nearby. Responses were given through the buttons on the two sides of the device, with one hand only at a time using the index and the middle finger. Hand images are taken from https://www.pexels.com/. (**B**) Block organization and combination of cue predictability levels for each of the six blocks in Experiment 1–2, and for each of the four blocks in Experiment 3. (**C**) Trial-by-trial changes in the probability estimate that the cue will be valid for visual (red bars) and tactile (blue bars) targets in a fully separate processing mode (*w* = 1, upper part) or in a fully average processing mode (*w* = 0.5, lower part), in a block with 30% cue predictability for visual targets and 70% for tactile targets, as derived from the Bayesian ideal observer model. Individual trials are depicted by colored dots (upper row: valid trials, lower row: invalid trials).

Stimuli were presented on an in-house built device to deliver visual and tactile stimuli at nearby locations (see Fig. 1A). For the tactile stimuli, a piezo-electric stimulation device was used (Piezostimulator, QuaeroSys, St. Johann, Germany), consisting of a control unit and two connected stimulation modules. Each module contained 24 pins arranged in a 4 × 6 matrix with the pins being 2.5 mm apart and covering an area of 7.5 mm × 12.5 mm. The pins could be moved (i.e., raised by 1.5 mm) independently. The stimulation units were positioned to touch the fingertip of both thumbs and the upper or lower row of pins was raised in trials with tactile targets. Visual stimuli were presented through four LEDs, two placed on the left side and two on the right side of the device (7.5° eccentric in each visual field), near the upper and lower row of pins of the tactile stimulator, along the thumb. A fifth LED delivering a constant dim red light was positioned at the center of the device, serving as fixation point.

A multisensory version of the Posner task^1^ was used. On each trial, a synthesized voice was presented binaurally over headphones pronouncing either “left” or “right” (“links” or “rechts” in German), acting as the spatial cue. The duration of the cue was 260 ms for left and 230 ms for right cues. After a 600 ms interval from the end of the auditory stimulus, either a visual or a tactile stimulus was presented for 100 ms. The intertrial interval was 2000 ms. The tactile stimulation consisted of a single movement of either the more proximal or the more distal row of pins, either on the left or on the right thumb. During visual stimulation one of the LEDs lighted up, either the proximal or the distal one with respect to the position of the thumb, either on the left or on the right side of the device. Participants were instructed to press a button with the index or middle finger of their right (or left) hand to indicate whether a distal or a proximal stimulus was presented. Participants responded with their right (or left, counterbalanced across participants) hand in the first half of the experiment, and with their left (or right) hand in the second half of the experiment. Therefore, the responding hand was independent from the location of the stimulation. Stimuli were controlled via an Arduino microcontroller (Arduino MEGA 2560 REV3, https://www.arduino.cc/), through Presentation software (Neurobehavioral Systems, Inc., Berkeley, CAR). Responses and reaction times (RTs) were registered using Brain Vision recorder (5 kHz sampling rate; Brain Products, Munich, Germany) to allow the recording of RTs with 1 ms precision^12^. In Experiment 2 and 3, a tone of 100 ms duration indicating the target modality was presented 460 ms before the spatial cue (see Fig. 1A, dashed frame). The two modalities were always correctly cued by two different pitches of the tone. The association between the modality and tone pitch was counterbalanced across participants.

In Experiment 1 and 2, six experimental blocks were presented to the participants (see Fig. 1B, upper part). The cue predictability was manipulated between blocks, but was kept constant within each block. Four different levels of cue predictability were used: 90%, 70%, 50%, and 30%. The predictability was different for visual and tactile targets within each block. In Experiment 3, the organization of the blocks changed (see Fig. 1B, lower part). Four experimental blocks were presented to the participants. Four different levels of cue predictability were used: 90%, 70%, 30%, and 10%. The cue predictability was different for the visual and tactile modality within each block, but the cue was equally predictive in both modalities when taking into account the difference from 50% (both target locations equally likely).

Throughout the experiments, participants were never informed about the levels of cue predictability, but they were aware that cue predictability could differ between the two modalities, so that one modality could be more predictable than the other in some of the blocks and vice versa, and that the levels of cue predictability might change between blocks. Each participant was presented with the same sequence of trials and the same blocks within each run. This is a standard procedure in computational studies of learning processes that require inference on conditional probabilities in time series.

Each block comprised 80 trials (40 trials per modality), resulting in a total of 480 trials in Experiments 1 and 2, and a duration of about 30 mins for each experiment. In Experiment 3, as only four blocks were presented, the total number of trials amounted to 320, for a duration of about 20 mins.

Short breaks of 20 sec were included at the end of each block with a tone indicating the beginning and the end of the break, and a longer break of 1 min was provided after the first half of the experiment. RTs and accuracy were recorded.

Each participant completed a short practice session at the beginning of the experimental session. During the practice two blocks of 40 trials each were presented. The first block contained 20 trials with only visual stimuli with 80% of cue predictability and another 20 trials with only tactile stimuli with 80% of cue predictability, whereas the second block contained mixed tactile and visual trials with 80% of cue predictability for each modality.

### Data analysis

For the RT analysis, incorrect trials, misses, anticipations (RT < 100 ms), and responses deviating more than 2 standard deviations from the individual subject’s mean were excluded from the analysis. For the analysis of accuracy, incorrect trials, misses, anticipations (RT < 100 ms), and responses deviating more than 2 standard deviations from the individual subject’s mean were considered as errors. The mean RT and the percentage of correct responses were analyzed to test for differences between experiments with independent samples t-tests and for differences between modalities with paired samples t-tests.

For the modelling approach, response speed (RS = 1/RT) was determined for each trial. RSs were used for the modeling because, in contrast to RTs, they are more normally distributed^13,14^.

In close analogy to the procedure described in Bestmann *et al*.^15^, we used a Bayesian ideal observer model in order to model the trial-wise learning of the modality-specific probabilistic contexts based on trial-wise RS.

For a discrete variable *x* that can take values from 1 to *K*, the vector *p* = *[p*_1_*, …p*_*K*_] parameterizes the probability distributions on *x*, with:

1 $$P(x=k)=\,{p}_{k}$$

and-for *N* samples of *x* - $X=\{{x}_{1},\,\ldots ,\,{x}_{N}\}$

2 $$P(X|p)=\prod _{k=1}^{K}{p}_{k}^{{N}_{k}}$$

*N*_*k*_ is the number of instances of the *k*-th trial type. Since our experiment comprised two events in each modality that impact on cue predictability (validly and invalidly cued targets), *K* equals 2 so that $P(X|p)$ in each modality follows a binomial distribution:

3 $$P(X=1)=\,p$$

4 $$P(X|p)={p}^{{N}_{1}}{(1-p)}^{{N}_{0}}$$

*N*_1_ is the number of valid trials and *N*_0_ is the number of invalid trials. The conjugate prior for the Bernoulli distribution is the Beta distribution (*Be*):

5 $$P(p)=Be(p|{\alpha }_{1,}{\alpha }_{0})$$

where *α*_0_ and *α*_1_ are hyper-parameters, acting as pseudo counts of the two trial types. The application of Bayes rule to derive the posterior distribution yields

6 $$\begin{array}{c}\begin{array}{rcl}P(p|X)\propto P(X|p)P(p) & = & P(X|p)Be(p|{\alpha }_{1},{\alpha }_{0})\\  & \propto  & [{p}^{{N}_{1}}{(1-p)}^{{N}_{0}}][{p}^{{\alpha }_{1}-1}{(1-p)}^{{\alpha }_{0}-1}]\\  & = & {p}^{{N}_{1}+{\alpha }_{1}-1}{(1-p)}^{{N}_{0}+{\alpha }_{0}-1}\\  & \propto  & Be(p|{\alpha }_{1}+{N}_{1},{\alpha }_{0}+{N}_{0})\end{array}\end{array}$$

Assuming a uniform prior (*α*_0_ = *α*_1_ = 1), i.e., that both events are equally likely at the beginning of each block, the mean of the posterior distribution, in our case parameterizing the expectation of a valid trial given *N* = *N*_1_ + *N*_0_ observations, is given by:

7 $$p=\frac{{N}_{1}+1}{{N}_{1}+{N}_{0}+2}$$

*p* was calculated separately for visual and tactile targets.

The integration of the resulting modality-specific probabilities was parameterized by the weighting factor *w* which was estimated from observed response speed (see below). In a given trial of an experimental block, the combined expectation *p*_*int*_ was a linear combination of the modality-specific probabilities with *p*_*mod1*_ being the probability of one modality (either visual or tactile) and *p*_*mod2*_ being the probability of the respective other modality (either tactile or visual):

8 $${p}_{int}=w{p}_{mod1}+(1-w){p}_{mod2}$$

with $$w\in \{0,1\}$$.

Values of *w* close to 0.5 reflect an equal weighting of both probabilities, i.e., a full integration (averaging) of modality-specific cue predictabilities. In contrast, values close to 1 indicate a separate processing of cue predictability in the two modalities. Figure 1C exemplarily depicts the model-derived trial-by-trial expectations in one experimental block for these two extremes. Values smaller than 0.5 reflect a larger weighting of the respective other modality.

To estimate the weighting parameter *w* for each modality, participant and experimental block, a response model was used to map the derived weighted probability estimates to the observed RSs. The response model assumes a linear relationship between trial-wise (*t*) response speed *RS*^*(t)*^ and the prediction before the observation of the outcome of the trial *t* which comes from the previous trial ${p}_{int}^{(t-1)}$ (see Vossel *et al*.^16^ for a similar procedure):

9 $$R{S}^{(t)}=\{\begin{array}{l}{\zeta }_{1\_{valid}}+{\zeta }_{2\_{valid}}\,{p}_{int}^{(t-1)}{\rm{for}}\,{\rm{valid}}\,{\rm{trials}}\\ {\zeta }_{1\_{invalid}}+{\zeta }_{2\_{invalid}}(1-{p}_{int}^{(t-1)})\,{\rm{for}}\,{\rm{invalid}}\,\mathrm{trials}\,\end{array}$$

${\zeta }_{1\_valid}$, ${\zeta }_{1\_invalid}$, ${\zeta }_{2\_valid}$, and ${\zeta }_{2\_invalid}$ are additional subject-specific parameters that are estimated from the data. While ${\zeta }_{1\_valid}$ and ${\zeta }_{1\_invalid}\,\,$determine the constants of the linear equation (i.e., the overall levels of RSs), ${\zeta }_{2}$ parametrizes the slope of the affine function (i.e., the strength of the increase in RS with increased estimated cue validity ${p}_{int}^{(t-1)}$).

Variational Bayesian estimation was used to derive the model parameter *w* and the response model parameters based on RSs separately for each modality, as implemented in the HGF toolbox (http://www.translationalneuromodeling.org/tapas/) running on MATLAB® (2012b, The MathWorks, Inc., Natick, Massachusetts, United States).

To test for the appropriateness of our modeling approach, we formally compared our Bayesian ideal observer model with an alternative model in which a constant level of predictability was fitted to each block. In order to have the necessary amount of trials to run a proper comparison between the two models, we concatenated the blocks for this analysis. For this control analysis, the log-model evidences resulting from the variational Bayesian model inversion of the competing models were compared using random-effects Bayesian Model Selection^17^ (BMS) for all blocks but separately for visual and tactile targets. Protected exceedance probabilities of the competing models are reported. The protected exceedance probability^18^ uses the Bayesian omnibus risk to compute a Bayesian model average of the exceedance probability, that is, the estimation of the likelihood of a particular model being the best compared with any other model, given the data. Importantly, random-effects BMS treats the model itself as being selected probabilistically by each subject in the population, enabling group-level inference while accounting for interindividual differences (e.g., the optimal model can vary across subjects).

The mean value of the weight parameter *w* over all experimental blocks was tested for differences between experiments with independent samples t-tests.

We hypothesized that the higher the cue predictability (i.e., the distance from 50%) and the higher the divergence between the two modality-specific probabilistic contexts, the easier it would be for the participants to rely on the respective cue predictability in this modality, therefore favoring a separate processing of modality-specific probabilistic contexts.

To test the impact of these two factors on the weighting parameter *w*, we analyzed the block-specific *w* values using the linear mixed-effects models (LMMs). The values of *w* for each participant in each condition (experimental block) and modality were the dependent variable. A stepwise procedure was followed, adding one at a time each factor and second-level interactions. The factors included were the *experiment* (only for Experiment 1 and 2), the *modality* (visual, tactile), the true *cue predictability* (calculated as absolute distance from 50%), and the *divergence* (i.e., the distance between the two modality-specific probabilistic contexts; e.g. 40 for a block with 90% and 50% true cue predictability). The models included also a random intercept for participants, to account for individual differences.

LMMs were performed using R (version 3.3.3; http://www.r-project.org/) and in particular using the lmer function (lme4 package; http://cran.r-project.org/web/packages/lme4/index.html). Models were compared using the “anova” function and factors (and interactions) were kept in the model only if they caused a significant increase of fit (tested by the Akaike Information Criterion, AIC). Reduced AIC was used as criterion for model selection because it favors parsimonious models, even in situations when the sample size is small^19^.

### Eye movement data recording

Eye-movements were monitored with an Eye-Link® 1000 (SR Research) eye-tracking system with a sampling rate of 500 Hz. At the start of the experiment, a calibration and validation of the eye-tracker were performed aiming at a validation error <1° of visual angle. Analysis of the data was performed using the ILAB toolbox^20^ in MATLAB (The MathWorks, Inc., Natick, Massachusetts, United States). The amount of time spent within a fixation zone of 1.5° from the central fixation point was analyzed for the time between cue and target appearance. Percentage of fixation time within the central ROI in the cue-target period was compared between visual and tactile targets with a paired samples t-test, and between experiments separately for visual and tactile targets with independent samples t-tests. Due to technical difficulties with eye-tracking owing to the use of the custom-made device without a screen, the signal quality was low in some datasets so that we analyzed only datasets with good signal in at least 50% of the trials. Therefore, we analyzed the data coming from seven participants in Experiment 1, 10 participants in Experiment 2, and 12 participants in Experiment 3.

### Data availability

The datasets generated during and/or analyzed during the current study are available from the corresponding author on reasonable request.

## Results

### Experiments 1–2: Integration is dependent on cue predictability and divergence

The same order of trials and the same combination of cue predictabilities were presented in the two experiments, allowing for a direct comparison. The two experiments differed only in the presence of the additional cue indicating the target modality in Experiment 2.

By trend, participants tended to be more accurate in Experiment 2 than in Experiment 1 (t(40) = 2.02, p = 0.05; Experiment 1: 90.6% ± 1.3%, SEM; Experiment 2: 93.6% ± 0.6%). The accuracy for visual and tactile targets did not differ significantly (Experiment 1: t(20) = 1.96, p = 0.06; Experiment 2: t(20) = 0.245, p = 0.81). Participants were not faster in their responses in Experiment 2, compared with Experiment 1 (t(40) = 1.56, p = 0.13; mean RT Experiment 1: 477 ± 17 ms; Experiment 2: 439 ± 17 ms). RTs for visual and tactile targets did not differ (Experiment 1: t(20) = 0.44, p = 0.67; Experiment 2: t(20) = −0.79, p = 0.44). The percentage of fixation time did not differ between visual and tactile targets (Experiment 1: t(6) = −1.94, p = 0.1, 94% fixation time for visual targets, 95% for tactile targets; Experiment 2: t(9) = −2.16, p = 0.06, 89% fixation time for visual targets, 91% for tactile targets). We also found no differences between experiments in fixation time neither for visual (t(15) = 0.94, p = 0.36) nor for tactile targets (t(15) = 0.97, p = 0.35).

The average value for the weighting parameter *w* did not differ significantly between Experiments 1 and 2 (t(40) = −0.56, p = 0.58; Experiment 1: 0.56 ± 0.2; Experiment 2: 0.58 ± 0.2).

**Figure 2:**
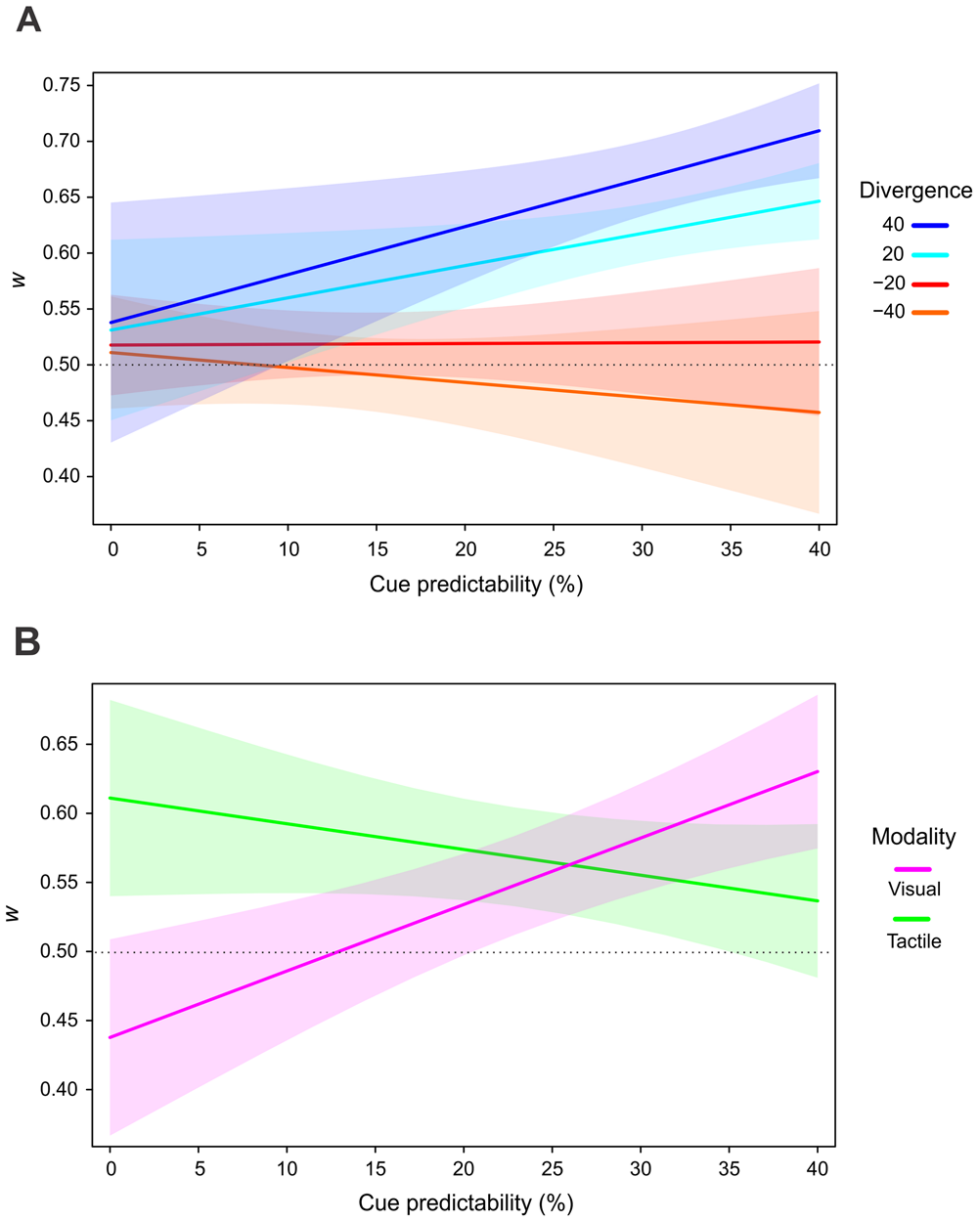
Results of the LMM on the weighting of cue predictabilities (*w* parameter) in Experiments 1–2. (**A**) Higher weighting of modality-specific probabilistic contexts in the context of positive divergence and high predictability. (**B**) Processing of visual and tactile stimuli is differentially sensitive to cue predictability. Colored areas represent the simulated 95% confidence interval of the coefficients.

LMM was used to test if the weighting of modality-specific cue predictability (i.e., the weighting parameter *w* in the different experimental blocks) was influenced by the modality of the stimuli, by the overall level of cue predictability, and/or by the divergence between the two cue predictabilities. Moreover, we tested if the experimental manipulation introduced in Experiment 2 changed the participants’ attentional processes. The best fitting LMM (see Fig. 2 and Table 1) showed that *w* values could be explained by the main effect of modality, by the interaction between *cue predictability* and *divergence*, and by the interaction between *cue predictability* and *modality*.

**Table 1:** Summary of the best fitting LMM for *w* values for Experiments 1–2.

Weighting factor *w*					95% CI	
*Fixed effects*	β	SEM	*t*	*p*	Lower	Upper
Intercept	0.61	0.04	16.9	**<0.001**	0.54	0.68
Cue predictability	−0.002	0.001	−1.4	0.16	−0.0045	0.0007
Divergence	0.0003	0.0008	0.44	0.66	−0.0012	0.0018
Modality *(visual)*	−0.17	0.04	−4.15	**<0.001**	−0.25	−0.09
Predictability x divergence	7.0e–05	4.3e–05	2.4	**0.02**	1.2e–05	0.0001
Predictability x modality	0.007	0.0015	4.3	**<0.001**	0.004	0.01
*Random effects*	SD					
Subjects (intercept)	0.01					
Residual	0.24					
AIC = −0.7						

β: beta estimate; SEM: standard error; CI: confidence interval; SD: standard deviation; AIC: Akaike Information Criterion. Significant *p* values are in bold. Reference condition for the categorical factor is reported in italic in brackets.

As shown in Fig. 2, *w* tended to deviate from 0.5 with higher positive divergence, i.e., when the cue predictability of the modality of the target considered was higher than the cue predictability of the other modality, and only when cue predictability itself was high. The effect was not present for the targets whose modality had a negative divergence. The factor *modality* interacted also with *cue predictability*, showing increasing *w* values with increasing cue predictability for the visual targets, and a tendency towards an inverted pattern for tactile stimuli. On the basis of the response model parameters, we calculated observed and predicted RS costs in the different experimental blocks. Figure S1 (for Experiment 1; see Supplementary materials) and Figure S2 (for Experiment 2) show that the observed RS pattern closely matched the RS pattern predicted by the Bayesian ideal observer model, corroborating the validity of our modelling approach.

Moreover, we compared our model with an alternative model in which a constant level of predictability was fitted to each block. In this model, there was no trial-by-trial learning of cue predictability. Instead, RSs for valid and invalid trials were constant in each block and depended on one constant level of cue predictability (estimated from the data). Random-effects BMS showed that our Bayesian learning model had higher model evidence than the alternative model with constant predictability, both for Experiment 1 and Experiment 2 (see Table 2).

**Table 2:** Results of the BMS model selection.

	Visual	Tactile
Model	PXP	PXP
*Experiment 1*		
Bayesian model	0.99	0.94
Constant model	0.01	0.06
*Experiment 2*		
Bayesian model	0.91	0.74
Constant model	0.09	0.26

PXP: protected exceedance probability.

The results revealed that the experimental manipulation introduced in Experiment 2, i.e., cueing also the target modality, did not significantly change participants’ behavior (i.e., there was no main effect or interaction with the factor *experiment*). When stimuli of different modalities were associated with different but concurrent probabilistic contexts, participants tended to merge the different cue predictabilities. However, two factors critically impacted on the degree of merging: the level of cue predictability of the target modality and the divergence between the two modality-specific probabilistic contexts. When the target had higher cue predictability compared with the target of the other modality (i.e., with higher positive divergence) and the level of cue predictability for the respective modality was high, values for *w* increased above 0.5, thereby reflecting a deviation from an averaging to a more separate processing of cue predictability.

### Experiment 3: Higher separate processing with modality-equal cue predictability and increased divergence

In the third experiment, different levels of cue predictability were employed and blocks combined only equally predictive cues for the two modalities, thereby increasing the divergence of the two probabilistic contexts (see Fig. 1B). Therefore, due to the different organization of the experimental protocol, these results had to be analyzed separately. Accuracy in Experiment 3 (94% ± 0.7%, SEM) did not differ from that observed in Experiment 2 (t(40) = 0, p = 1) or Experiment 1 (t(40) = 1.98, p = 0.055), and the accuracy for visual and tactile targets did not differ significantly (t(20) = −1.23, p = 0.23). However, RTs in Experiment 3 (420 ± 11 ms) were faster than those observed in Experiment 1 (t(40) = 2.8, p = 0.007). Nevertheless, RTs did not differ from those observed in Experiment 2 (t(40) = 0.93, p = 0.36). In Experiment 3 RTs for visual and tactile targets did not differ (t(20) = 0.37, p = 0.72). The percentage of fixation time did not differ between visual and tactile targets (t(11) = −1.97, p = 0.075, 88% fixation time for visual targets, 89% for tactile targets). We also found no differences between Experiment 3 and Experiments 1 and 2 in fixation time, neither for visual (compared with Experiment 1: t(17) = 1.62, p = 0.12; compared with Experiment 2: t(20) = 0.24, p = 0.81) nor for tactile targets (compared with Experiment 1: t(17) = 1.73, p = 0.1; compared with Experiment 2: t(20) = 0.27, p = 0.79).

The values of the weighting parameter *w* in Experiment 3 (0.67 ± 0.02) were significantly higher than in Experiment 1 (t(40) = −3.36, p = 0.002) and in Experiment 2 (t(40) = −2.72, p = 0.01), reflecting a higher degree of separation of the two probability contexts.

The best fitting LMM on *w* included only the main effect of *modality*, with higher *w* values for visual (mean = 0.67, 95% CI [0.63 0.71]) than tactile targets (mean = 0.61, 95% CI [0.56 0.65]). The factors *cue predictability* and *divergence* did not significantly increase the fit of the model. Table 3 provides the descriptive data of the single parameters and the levels of significance. Figure S3 (see Supplementary materials) shows the observed and predicted RS cost patterns from the response model.

**Table 3:** Summary of the best fitting LMM for *w* values for Experiment 3.

Weighting factor *w*					95% CI	
*Fixed effects*	β	SEM	*t*	*p*	Lower	Upper
Intercept	0.60	0.02	27.2	**<0.001**	0.56	0.65
Modality (*visual*)	0.06	0.03	2.1	**0.04**	0.004	0.12
*Random effects*	SD					
Subjects (intercept)	0.04					
Residual	0.19					
AIC = −68.3						

β: beta estimate; SEM: standard error; CI: confidence interval; SD: standard deviation; AIC: Akaike Information Criterion. Significant *p* values are in bold. Reference condition for the categorical factor is reported in italic in brackets.

Random-effects BMS showed that our Bayesian ideal observer model had higher model evidence than the alternative model with constant predictability, both for Experiments 1 and 2 (see Table 4).

**Table 4:** Results of the BMS model selection.

Model	Visual	Tactile
	PXP	PXP
*Experiment 3*		
Bayesian model	0.82	0.59
Constant model	0.18	0.41

PXP: protected exceedance probability.

In Experiment 3 the cue was equally predictive for both modalities (albeit in opposite directions). As a consequence, also the level of divergence of the two modality-specific probabilistic contexts was increased, compared with Experiments 1 and 2. Our hypothesis was that under these circumstances participants would use a more separate processing of the probabilistic contexts. Indeed, the values of the weighting parameter *w* were generally higher than in Experiments 1 and 2, suggesting that participants were assigning higher weights to the respective modality-specific cue predictability. In addition, in the context of equal predictability of the cue and higher divergence for the two modalities, the magnitude of the weighting parameter *w* neither depended on the level of cue predictability nor on the divergence between the two probabilistic contexts. We found that the *w* values were higher for the visual than for the tactile modality.

## Discussion

We investigated the deployment of spatial attention in response to visual and tactile stimuli throughout multiple behavioral experiments. The location of visual and tactile stimuli was cued on a trial-by-trial basis with varying levels of predictability. Two different and concurrent probabilistic contexts were associated with the two modalities, by assigning different levels of cue predictability to visual and tactile targets. By estimating the degree of integration of both probabilistic contexts (parameterized by *w*) in the different experimental blocks, we tested whether participants processed the two probabilistic contexts independently, or whether they merged the two cue predictability levels into a combined (average) expectancy. We also tested if this merging is flexible, i.e., whether it is modulated by the level of cue predictability or by the divergence between both cue predictabilities.

In Experiment 1 participants were not informed about the modality of the upcoming target. Therefore, they were required to prepare for both target modalities in each trial, whereas in Experiment 2 the modality of the upcoming target was specified in advance. Results suggested that the additional information concerning the target modality did not significantly change participants’ behavior. The data showed that although a merging of modality-specific contexts occurred, the degree of merging was flexible and depended on an interactive effect of cue predictability and divergence, with more separate processing of modality-specific probabilistic contexts when cue predictability was high and with positive divergence. One could have expected that presence of the additional cue indicating target modality in Experiment 2 would induce a more separate processing of the two modality-specific probabilistic contexts. However, the results did not point to this direction. This may indicate that the merging does not occur at the stage of orienting towards a visual or tactile target, but rather at the stage of the estimation of cue predictability for attentional orienting. In Experiment 3, the values of the weighting factor were generally higher than in Experiments 1 and 2, indicating a higher degree of separate processing of the modality-specific probabilistic contexts. In this experiment, the degree of cue predictability for the two modalities was comparable, and the divergence of the two probabilistic contexts was increased. Given the results of Experiments 1 and 2, it is likely that both factors contributed to the increase in the degree of separation in the processing. Based upon the results of Experiment 3, we conclude that cue predictability and divergence of the probabilistic contexts are factors that modulate the degree of merging of the modality-specific probabilistic context to a stronger extent, more than the presence of information concerning the target modality.

In addition, the weighting was generally higher for visual than for tactile stimuli. This may be due to a different experience with processing visual or tactile external stimuli to predict their location, making the visual information more precise and therefore favoring a higher degree of separate processing.

Using a very similar multisensory location-cueing paradigm, Spence *et al*.^8^ performed a series of behavioral experiments to explore crossmodal links in spatial attention during visual and tactile stimulation. In their Experiment 4 they manipulated participants’ attention by asking them to direct their attention mainly to one modality (i.e., the primary modality) while keeping the attention for the other modality (i.e., the secondary modality) diffused. One side of space served as the most probable location of the target in the primary modality. The probability of target occurrence was manipulated, so that more targets overall appeared in the primary modality and more frequently on the attended side. As expected, they showed faster detection for targets that appeared in the attended side. In contrast, even though targets of the secondary modality appeared more frequently on the side opposite to the one attended for the primary modality, detection was also faster for these targets when occurring on the attended side in the primary modality. In other words, spatial expectancies in one modality modulated expectancies in the other modality, independently from the probability of occurrence of the latter. However, the size of the cueing effect was greater in the primary than in the secondary modality, suggesting that crossmodal connections in attentional systems between modalities do exist, but at the same time arguing against a fully supramodal account of attention.

In our study, the computational modeling approach and the systematic manipulation of cue predictability levels allowed us to directly quantify the degree of merging of modality-specific cue predictability and to identify factors that impact upon the degree of integration. Our results therefore provide a formal computational explanation of the previous findings by Spence and colleagues. In line with the data from Spence *et al*.^8^, a purely modality-specific (i.e., separate) processing was not present in all conditions, as average values of the weighting factor *w* never approached 1. The magnitude of the weighting parameter depended on cue predictability and the divergence between the modality-specific probabilistic contexts, as well as the task structure. Accordingly, the present findings are in line with the “separable-but-linked” hypothesis^7^, suggesting the presence of separable spatial attentional systems that are usually connected and work in concert unless circumstances induce us to allocate spatial attention to different modalities independently (such as a different cue-related reliability in the different modalities as in the present study). Additional evidence from studies on multimodal integration also point to a flexible weighting of the information from different modalities, depending on the precision of the modality-specific information^21,22^. Within visuo-tactile integration, when participants had to judge the height of two ridges relying on both tactile and more-or-less noisy visual information, the weights assigned to the two modalities in order to perform the estimate critically depended on the amount of noise in the visual information^21^.

A similar process applied to visuo-auditory integration^22,23^. When participants had to guess the location of concurrent auditory and visual stimuli, the visual information, in itself more precise than the auditory one in its spatial information, biased the judgment about the location of auditory stimuli^23^, and this bias was stronger when the precision of the visual information was increased^22^. Also in our present results, when cue predictabilities were matched between modalities, visual stimuli induced a higher degree of separate processing, compared with the tactile modality.

In conclusion, our data suggest that the processing of concurrent modality-specific probabilistic contexts during a spatial attention paradigm is based on a flexible integration of modality-specific expectancies, depending on factors such as the degree of predictability and the divergence between the modality-specific predictabilities. These results motivate studies using neuroimaging or neurophysiological techniques to identify the neural signatures of this highly flexible and adaptive processing.

## Electronic supplementary material

Supplementary materials
