# Supplementary material for: Integrating modality-specific expectancies for the deployment of spatial attention
Source: Sci Rep. 2018 Jan 19;8:1210. doi: 10.1038/s41598-018-19593-7 (PMC5775425; doi:10.1038/s41598-018-19593-7)

Integrating modality-specific expectancies for the deployment of spatial attention

Paola Mengotti[1,*], Frank Boers[2], Pascasie L. Dombert[1], Gereon R. Fink[1,3], Simone Vossel[1,4]

[1]Cognitive Neuroscience, Institute of Neuroscience & Medicine (INM-3), Research Centre Juelich, 52425 Juelich, Germany

[2]Institute of Neuroscience & Medicine (INM-4), Research Centre Juelich, 52425 Juelich, Germany

[3]Department of Neurology, University Hospital Cologne, 50937 Cologne, Germany

[4]Department of Psychology, University of Cologne, 50923 Cologne, Germany

Supplementary materials

Figure S1. Experiment 1: observed and predicted pattern of RS costs from the Bayesian ideal observer model with weighting factor. RS costs were calculated by subtracting RSs of invalid trials from valid trials and are shown in relation to the participants' estimated probability that the cue will be valid in an upcoming trial $P^{(t-1)}$, binned in intervals of 0.2. As expected, RS costs increased with increased subjective level of cue predictability, and the pattern of observed RS costs matched the RS costs predicted by the model on the basis of individual values for ζ_{1_valid}, $\zeta_{1\_invalid}$, ζ_{2_valid}, and $\zeta_{2\_invalid}$ in the different blocks for visual and tactile targets. Error bars indicate SEM.

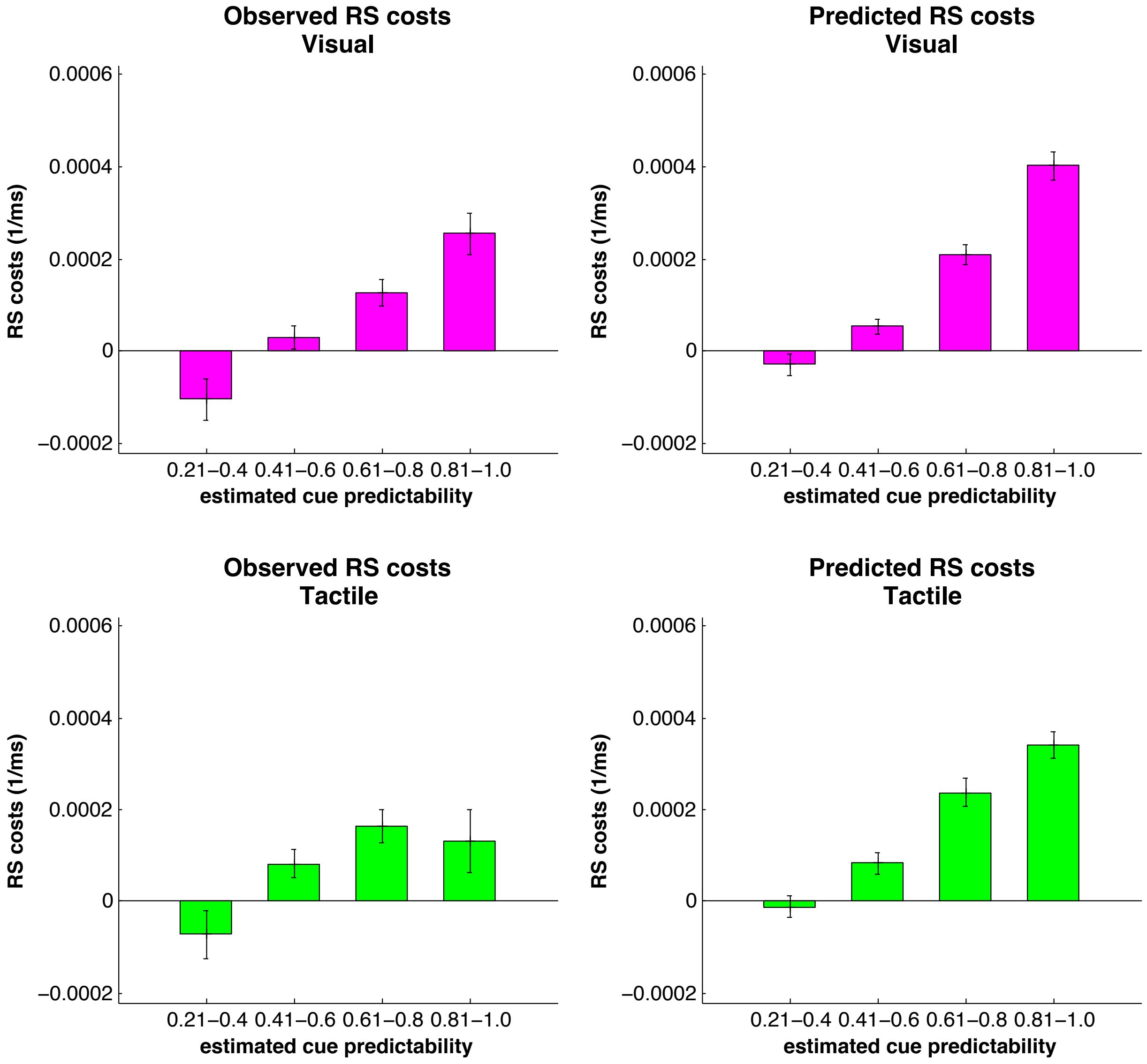

Figure S2. Experiment 2: observed and predicted pattern of RS costs from the Bayesian ideal observer model with weighting factor. RS costs were calculated by subtracting RSs of invalid trials from valid trials and are shown in relation to the participants' estimated probability that the cue will be valid in an upcoming trial $P^{(t-1)}$, binned in intervals of 0.2. As expected, RS costs increased with increased subjective level of cue predictability, and the pattern of observed RS costs matched the RS costs predicted by the model on the basis of individual values for ζ_{1_valid}, $\zeta_{1\_invalid}$, ζ_{2_valid}, and $\zeta_{2\_invalid}$ in the different blocks for visual and tactile targets. Error bars indicate SEM.

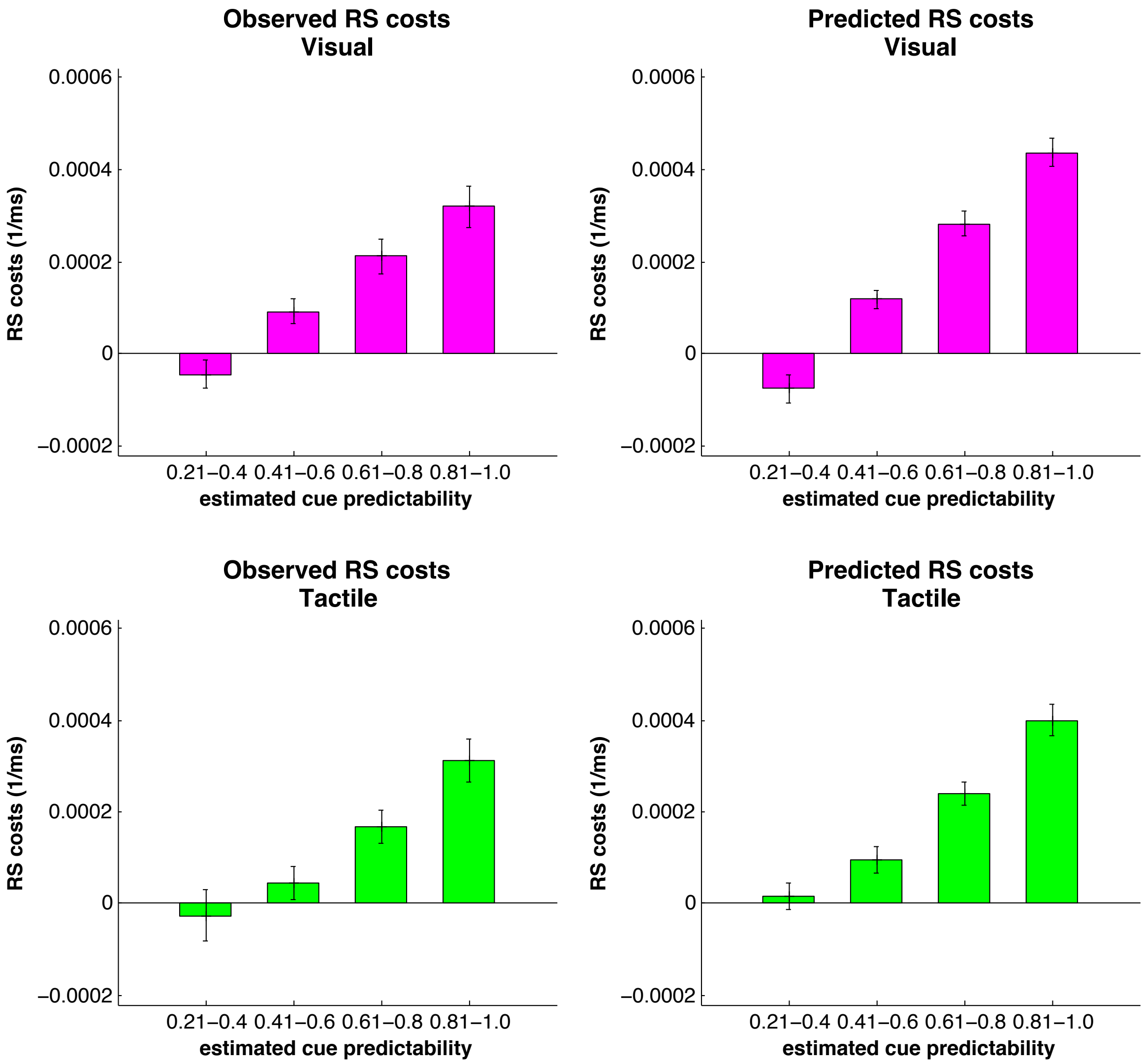

Figure S3. Experiment 3: observed and predicted pattern of RS costs from the Bayesian ideal observer model with weighting factor. RS costs were calculated by subtracting RSs of invalid trials from valid trials and are shown in relation to the participants' estimated probability that the cue will be valid in an upcoming trial $P^{(t-1)}$, binned in intervals of 0.3 (due to the different organization of the blocks). As expected, RS costs increased with increased subjective level of cue predictability, and the pattern of observed RS costs matched the RS costs predicted by the model on the basis of individual values for ζ_{1_valid}, $\zeta_{1\_invalid}$, ζ_{2_valid}, and $\zeta_{2\_invalid}$ in the different blocks for visual and tactile targets. Error bars indicate SEM.

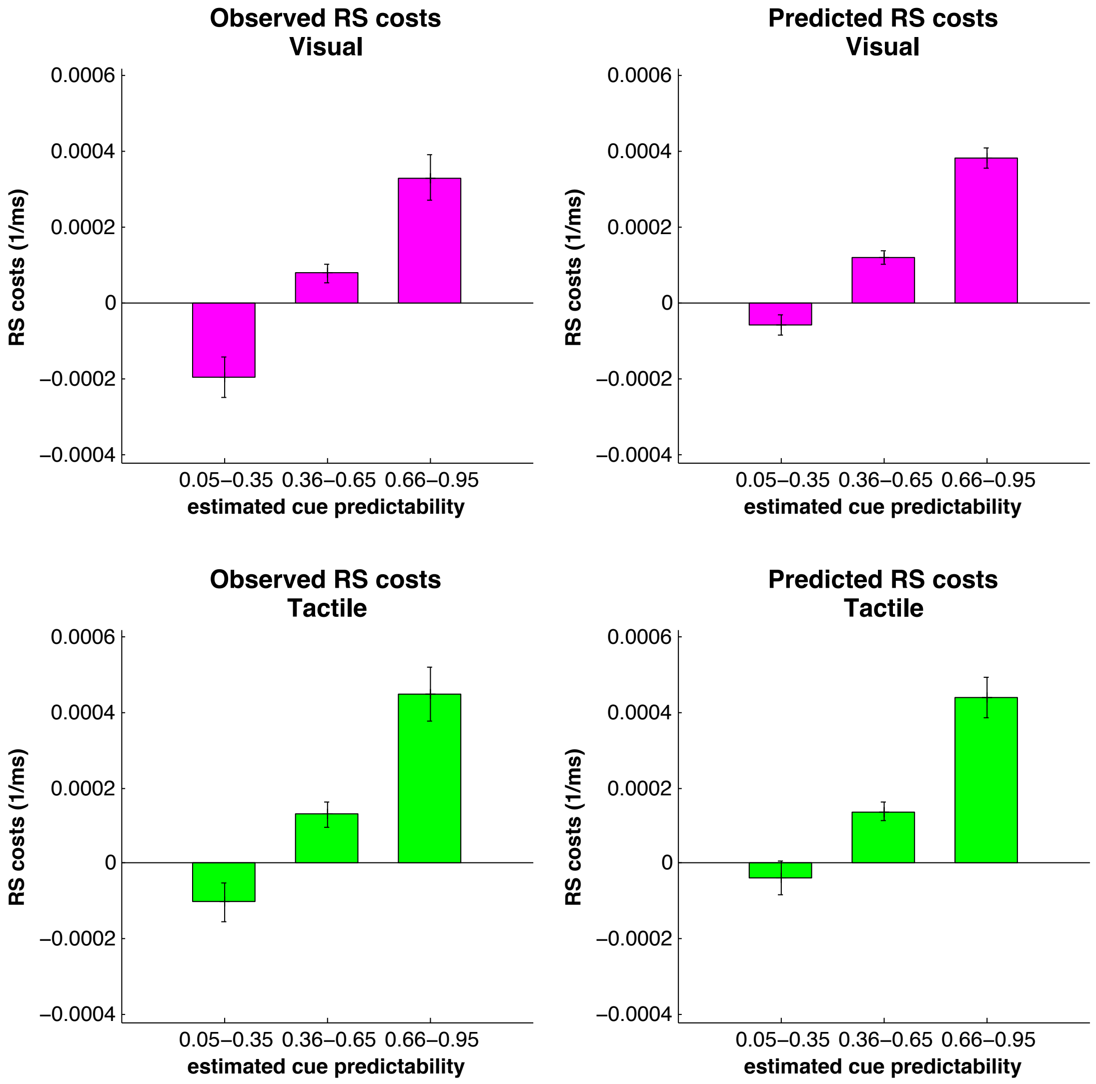

Supplement: Supplementary file 1 — Supplementary materials [file 41598_2018_19593_MOESM1_ESM.pdf]
